# A Study on Dissimilar Friction Stir Welded between the Al–Li–Cu and the Al–Zn–Mg–Cu Alloys

**DOI:** 10.3390/ma11071132

**Published:** 2018-07-04

**Authors:** Pengfei Wu, Yunlai Deng, Shitong Fan, Hua Ji, Xinming Zhang

**Affiliations:** 1 School of Materials Science and Engineering, Central South University, Changsha 410083, China; pfwu_csu@csu.edu.cn (P.W.); fanstone95@163.com (S.F.); xnixrd@126.com (H.J.); xmzhang@csu.edu.cn (X.Z.); 2 Nonferrous Metal Oriented Advanced Structural Materials and Manufacturing Cooperative Innovation Central, Central South University, Changsha 410083, China

**Keywords:** friction stir weld (FSW), the Al–Zn–Mg–Cu alloy, the Al–Li–Cu alloy, microstructure, diffusion, corrosion

## Abstract

In this paper, the microstructure of friction stir welded between the Al–Li–Cu alloy and the Al–Zn–Mg–Cu alloy was studied using optical microscope(OM), electronic backscattered diffraction (EBSD), SEM, and TEM. The hardness profile revealed that the range of heat affect zone for the Al–Zn–Mg–Cu alloy was slightly wider than the Al–Li–Cu alloy when they suffered from the thermal transient. Additionally, the characterization of precipitates inside the different zones was obviously different, which corresponded to the microhardness distribution profile. At the periphery of the kissing line, the Al–Li–Cu and the Al–Zn–Mg–Cu alloys mutually diffused during the weld process. The magnesium element in the Al–Zn–Mg–Cu alloy diffused into the Al–Li–Cu alloy. But the copper and zinc had no change because of the low diffusion coefficient in aluminum. The heat affected zone of the Al–Zn–Mg–Cu alloy showed a higher corrosion susceptibility immersed into the corrosion environment, and the Al–Li–Cu alloy did not show severe corrosion.

## 1. Introduction

Nowadays, with the development of industry and population growth, the energy crisis has been serious. Whether commercial or military aircraft, all of them pursue being lightweight to decrease energy consumption. Aluminum is widely applied to the aeronautical area because of its outstanding performance. Among all of the series of aluminum alloys, the Al–Zn–Mg–Cu alloys and Al–Li–Cu alloys occupy the major proportion. In the near future, we must solve the problem of connection between those two alloys.

The Al–Li–Cu alloys are celebrated for their high strength-to-weight ratio. Lithium is the pivotal element for the Al–Li–Cu alloys, compared with conventional Al–Cu alloys, as only 1% (wt %) of the Li being dissolved into the aluminum matrix will reduce the density by 3% and increase the elastic module by 6% [1]. This make it more competitive than conventional Al alloys for applications. Meanwhile, through the ageing process, the T_1_(Al_2_CuLi) phase containing lithium atoms provides a primary strengthening effect. In the Al–Li–Cu alloys system, the types of precipitates are dependent on the different composition and ageing conditions, excluding the T_1_(Al_2_CuLi) phase, the Guinier–Preston (GP) zones and the δ (AlLi), δ′ (Al_3_Li), θ′ (Al_2_Cu), S′ (Al_2_CuMg), and β′ (Al_3_Zr) phases, may precipitate [2,3,4,5]. Recent works revealed that the T_1_ precipitates with an anodic potential, contrasted to the Al matrix. Especially if the alloy is not a T8 condition, the corrosion potential of the matrix is different from the grain boundaries as a result of the inhomogeneous distribution of the T_1_ precipitates [6,7,8].

Similarly, the Al–Zn–Mg–Cu alloys are one of most popular aluminum alloys in the field of aircraft manufacturing, as it possesses a super high strength and low density, however only a little higher than the Al–Cu–Li alloys [9]. They are widely used to manufacture the envelope and framework of airplanes. The addition of the zinc and magnesium elements provides dense coherent η′ phase (MgZn_2_) precipitates inside grains, during the subsequent ageing, which makes them excellent for strength and fatigue properties [10,11]. However, they show a poor corrosion resistance without deliberate protection.

In the manufacturing processes of airplanes, there are many ways to combine Al–Li–Cu alloys with Al–Zn–Mg–Cu alloys, including riveting, bolted connection, and welding. Welding is one of the ideal technologies, without the weight addition of an aerospace material, so far. It is significant for FSW taking place the arc welding and riveting for light-weight engineering of aerospace. Both of the two types of high-strength aluminums are difficult to weld using traditional welding techniques. Friction stir weld (FSW) is a new solid welding technique used to weld the poor weldability metals and dissimilar metals that are difficult to weld together. FSW was invented by The Welding Institute (TWI), U.K., in 1991 [12]. The FSW joint consists of the nugget zone (NZ), thermomechanical affect zone (TMAZ), and heat affect zone (HAZ) [13], and there are discrepant microstructures in different zones.

FSW of similar aluminum alloys has been developed by many researchers. A.K. Shukla et al. [14] and Su et al. [15] separately used transmission-electron microscopy (TEM) to research the microstructural evolution during the FSW of the Al–Cu–Li alloy joint and the Al–Zn–Mg–Cu alloy (7050-T651) joint. The distributions of grain structure, the low-angle grain boundaries, and precipitate condition at different weld zones were investigated. Each region went through a different thermo-mechanical cycle during the FSW process, which was one of the major causes leading each region to possess a specific microstructure. Likewise, previous work also focused on the properties and microstructures of some dissimilar FSW aluminum alloys. M.M.Z. Ahmed et al. [16] studied the influence of tool traverse speed on the joint microstructure and strength of FSW, of which AA7075 was dissimilar to AA5083. Their result illustrates that both NZ grains, size AA7075 and AA5083, could be refined by increasing the traverse speed, but AA5083 had a coarser grain structure, and various traverse speeds brought out the disparity of the dissimilar joint strength. S Serajzadeh et al. focused on the microstructure, effect of tool geometry, residual stresses, and temperature field simulation regarding the dissimilar FSW 5086/6061 [17,18,19,20]. Furthermore, other dissimilar FSW aluminum alloys also were investigated, such as the AA2024/AA7075 couple [21] and the AA6061/AA7050 couple [22].

Both the Al–Li–Cu alloys and Al–Zn–Mg–Cu alloys are significant aeronautical structural materials. This article aims at researching a new connection between the two types of aeronautical materials, and it may be beneficial for the application of aluminums in aerospace field. It is significative for the development of the aviation industry to research the connection ways between two types of alloys. In addition to the strength, the corrosion resistance is necessary for aluminum alloys. Therefore, this research aims at recognizing the microstructural characterization and corrosion behavior of dissimilar friction stir welds to the Al–Cu–Li alloy and Al–Zn–Mg–Cu alloy. The corrosion result reveals the relationship between the microstructure and corrosion susceptibility, the Al–Zn–Mg–Cu alloy plays a decisive role in the integral corrosion test, to protect the Al–Li–Cu alloy.

## 2. Materials and Methods

**Figure 1:**
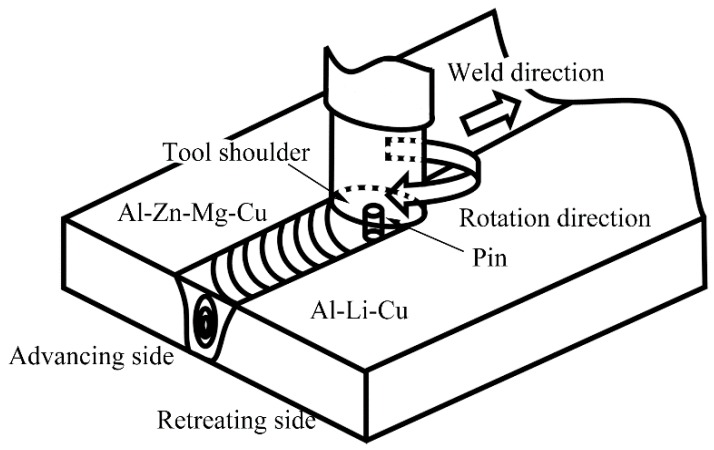
Schematic diagram of the friction-stir welding of the dissimilar Al–Li–Cu alloy to the Al–Zn–Mg–Cu alloy.

The 10 mm thick plates of the Al–Li–Cu alloy and Al–Zn–Mg–Cu alloy were friction stir welded by the FSW instrument with a tilt angle of 2.5°. The nominal rotation speed and traverse speed were 600 rpm and 300 mm/min. The welding direction was parallel to the rolling direction of the plates. The dissimilar FSW, the Al–Li–Cu alloy to the Al–Zn–Mg–Cu alloy, is schematically illustrated in Figure 1. The nominal chemical compositions in the weight percentage were Al(3.5)–Cu(0.9)–Li(0.36)–Mg(0.35)–Ag(0.35)-Mn(0.35) and Al(5.2)–Zn(3.0)–Mg(1.0)–Cu(1.0)–Zr(0.1). Both of the alloys were tempered at T6.

The optical microstructure of the FSW joint and the intergranular corrosion depth on the cross-section were observed by an OLYMPUS BX51M optical microscope. The specimens for the metallographic examination, taken from a cut-off cross-section of the weld, sandpaper, and soft cloth, were used to polish the specimens to be mirror-like without any contamination. Then, the sample was etched for 20 s using Keller’s etchant (2 mL HF, 5 mL HNO_3_, 3 mL HCl, and 190 mL of distilled water) for the optical microscopic observation. Electronic backscattered diffraction (EBSD) and an elements line scanning observation were conducted on a ZEISS EVO M10 scanning electron microscope, and the samples for EBSD and SEM were prepared like the specimens for the metallographic examination, then the sample for EBSD carried out electrochemical polishing at a 20 V.

The Vickers micro-hardness test was implemented by the Huayin hardness testing machine after 30 days of nature ageing (load = 300 gf, dwell time = 15 s, and spacing between each test point = 1 mm) on the center of the joint cross-section, perpendicular to the welding direction.

The exfoliation corrosion (EXCO) test was taken according to ASTM G34-2001 (2007). The samples for the test were ground with 2000 sandpaper, and were then polished. The EXCO test was conducted in a solution (4 mol/L NaCl + 0.5 mol/L KNO_3_ + 0.1 mol/L HNO_3_) at 25 °C for 96 h, in a water bath thermostat, and the ratio of the solution volume to surface of the specimens is approximate to 20 mL/cm^2^. The electrochemical tests were carried out using an electrochemical workstation (chi660e), and an electrochemical cell with a three-electrode containing a 3.5% NaCl solution was used to measure the open circuit potential (OCP) of the different weld zones.

The foils for the transmission electron microscope (TEM) observation were cut from the zones of the alloys that were suffered to different thermal cycles after nature ageing for 45 days. Transverse metallographic specimens were prepared so as to separate the various weld zones, and 3 mm diameter discs at a 100 μm were prepared for twin jet electro-polishing, with a solution of 25% HNO_3_, in methanol at a voltage of 20 V and a temperature of −20 °C. The TEM observation was carried out on a Tecnai G2 F20 microscope at an accelerate voltage of 200 KV.

## 3. Results

### 3.1. Microstructure

**Figure 2:**
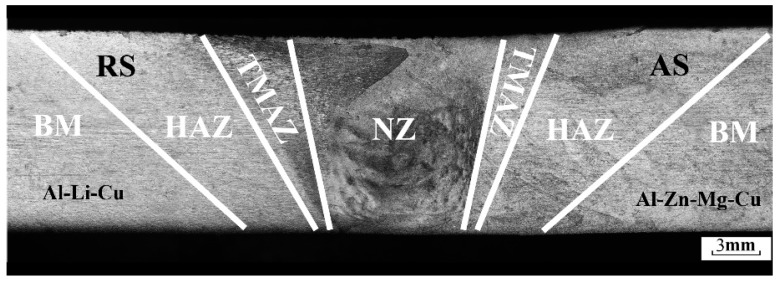
Macrostructure of the cross sections joint, advancing side (AS) is the Al–Zn–Mg–Cu alloy and retreating side (RS) is the Al–Li–Cu alloy. HAZ—heat affect zone; TMAZ—thermomechanical affect zone; NZ—nugget zone.

Figure 2 provides the cross-section optical macrostructure photography of the FSW joint. The Al–Zn–Mg–Cu alloy is located on the advancing side (AS) and the Al–Li–Cu alloy is located on the retreating side (RS). The FSW joint is shown in Figure 2. Different weld zones were suffered from the different degree of thermal cycle and mechanical stir during the welding process. No distinct welding defects are detected.

**Figure 3:**
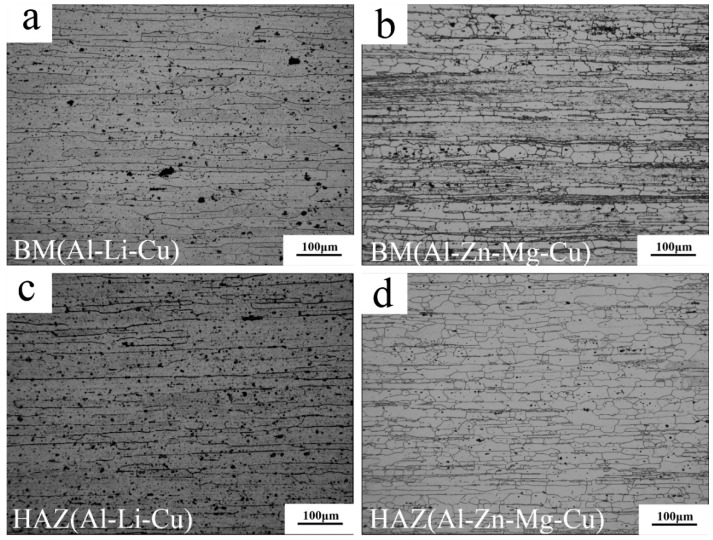

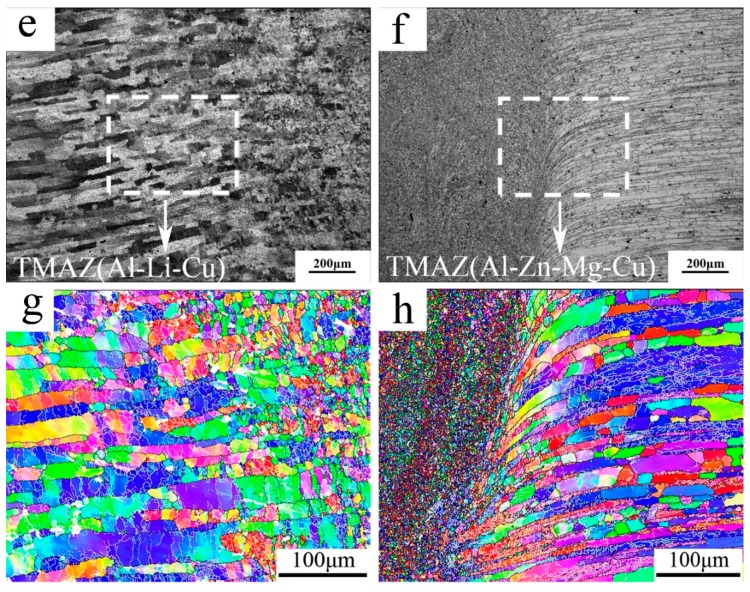
Optical microstructure of joint: (**a**) base material of the Al–Li–Cu alloy; (**b**) base material of the Al–Zn–Mg–Cu alloy; (**c**) HAZ of the Al–Li–Cu alloy; (**d**) HAZ of the Al–Zn–Mg–Cu alloy; (**e**) TMAZ of the Al–Li–Cu alloy at retreating side; (**f**) TMAZ of the Al–Zn–Mg–Cu alloy at the advancing side; (**g**) electronic backscattered diffraction (EBSD) image of TMAZ at the RS; and (**h**) EBSD image of TMAZ+NZ at AS.

**Figure 4:**
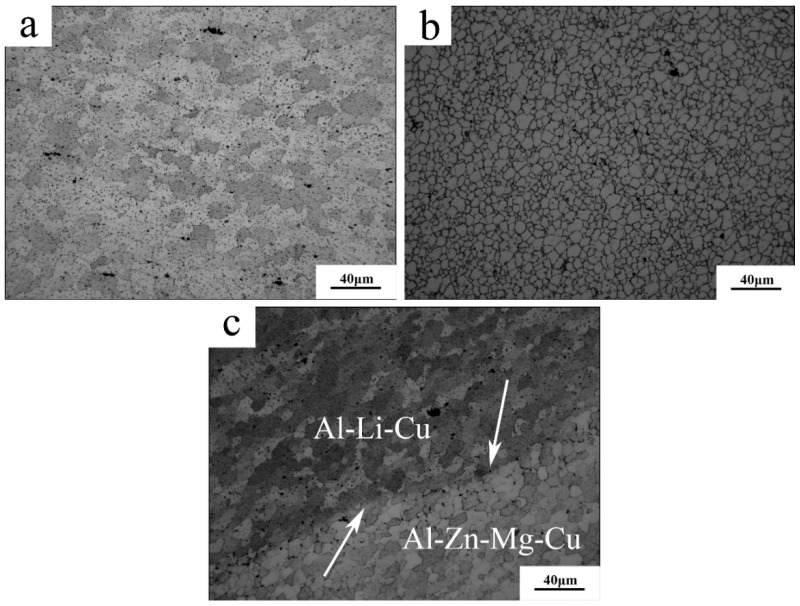
Optical microstructure of NZ: (**a**) NZ of the Al–Li–Cu alloy; (**b**) NZ of the Al–Zn–Mg–Cu alloy; and (**c**) kissing line.

The optical microstructure pictures for each of the typical zones are presented in Figure 3 and Figure 4. For the Al–Li–Cu alloy, the grains of base material (BM) (Figure 3a) and HAZ (Figure 3c) were similar, both were elongated along the transverse direction. The grains within TMAZ were distorted because of the pin stirring, and a number of grains were broken (Figure 3e). NZ (Figure 4a) comprised of fine recrystallized grains with an approximate 10–15 μm diameter. For the Al–Zn–Mg–Cu alloy, NZ showed ultra-fine grains with less than a 10 μm diameter (Figure 4b). Extremely fine grains were the result of friction heat and severe plastic deformation, and the Zr addition into the Al–Zn–Mg–Cu alloy inhibited the grain boundary removing [23,24] NZ, which shows finer grains. The grains within the BM and HAZ of the Al–Zn–Mg–Cu alloy also are elongated along the transverse direction. The percentage of the black area in BM (Figure 3b) is larger than that in HAZ (Figure 3d), illustrating there is a low degree of recrystallization in BM. There is a dramatic difference between the TMAZ of the advancing side and that of the RS, although the TMAZ of AS consist of the elongated distorted grains as well, a distinct border between the TMAZ and NZ at the AS was detectable. By approaching the mixed zone, as shown in Figure 4c, the Al–Zn–Mg–Cu alloy metallurgically integrated with the Al–Li–Cu alloy with an interface, pointed out with the white arrow.

Figure 3g,h portrays the EBSD images for the TMAZ of AS and RS. The white line represents the subgrain boundaries and the black line represents the grain boundary. At the AS, some smaller grains without deformation may recrystallize, which is the result of more heat input, because of the rare low-angle grain boundaries that exist inside those grains, but recrystallization was not detected at the RS.

### 3.2. TEM Observation

**Figure 5:**
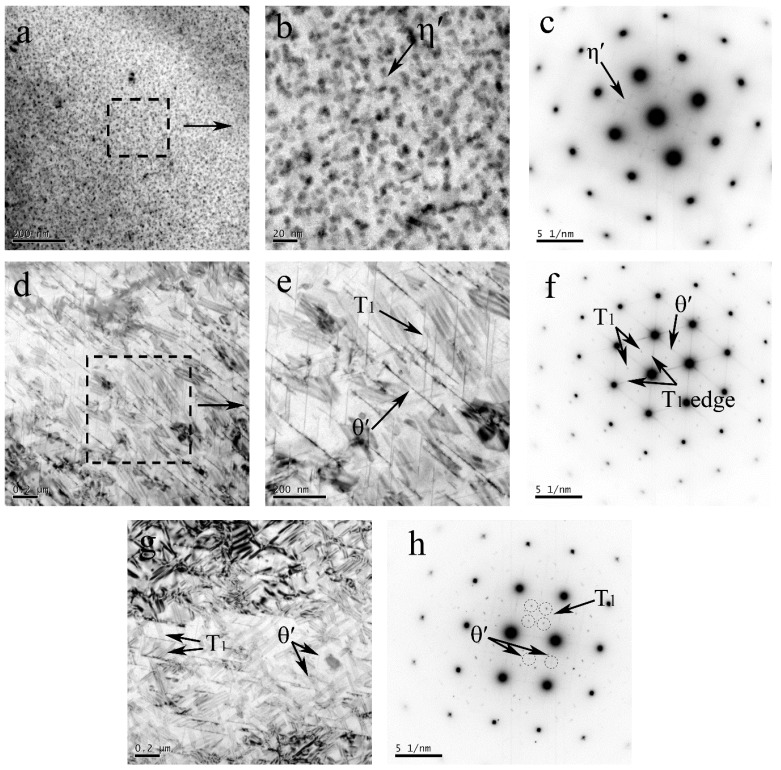
TEM images of inside grains for the different zones of the Al–Zn–Mg–Cu alloy at AS: bright field (BF)-images of (**a**) BM and (**b**) high magnification of BM; (**c**) selected area diffraction (SAD) pattern of BM along the [100]_Al_ axis zone; and for the different zones of the Al–Li–Cu alloy at RS: bright field of (**d**) BM and (**e**) high magnification of BM, (**f**) SAD pattern of BM along [110]_Al_ axis zone, (**g**) bright field of BM, (**h**) SAD pattern of BM along [100]_Al_ axis zone.

In the base materials of the Al–Zn–Mg–Cu alloy, the metastable η′ phase was coherent with the Al matrix; it effectively prohibits dislocation by moving to strength materials. The selected area diffraction (SAD) pattern (Figure 5c) in the [100]_Al_ zone axis illustrated that the intragranular precipitates were η′ phase, and that the bright field (BF)-TEM images (Figure 5a,b) presented uniformly distributed dense fine η′ phases with a size less than 10 nm. In the BM of the Al–Li–Cu alloy, the strong streaks, indicated by the black arrows in the SAD pattern (Figure 5f) taken along [110]_Al_ zone axis, revealed the presence of θ′ and T_1_ precipitates, and the bright field (BF) TEM images (Figure 5d,e) showed the T_1_ precipitates with a diameter of no more than 200 nm. The T_1_ precipitates are the dominant strengthening precipitate in the base material of the Al–Cu–Li alloy. Figure 5g is the BF images for the Al–Li–Cu alloy along the [100]_Al_ axis zone, which was consistent with the SAD pattern (Figure 5h). It reveals that some θ′ phase precipitates are present in BM, and that these θ′ precipitates range from 200 to 300 nm in length.

**Figure 6:**
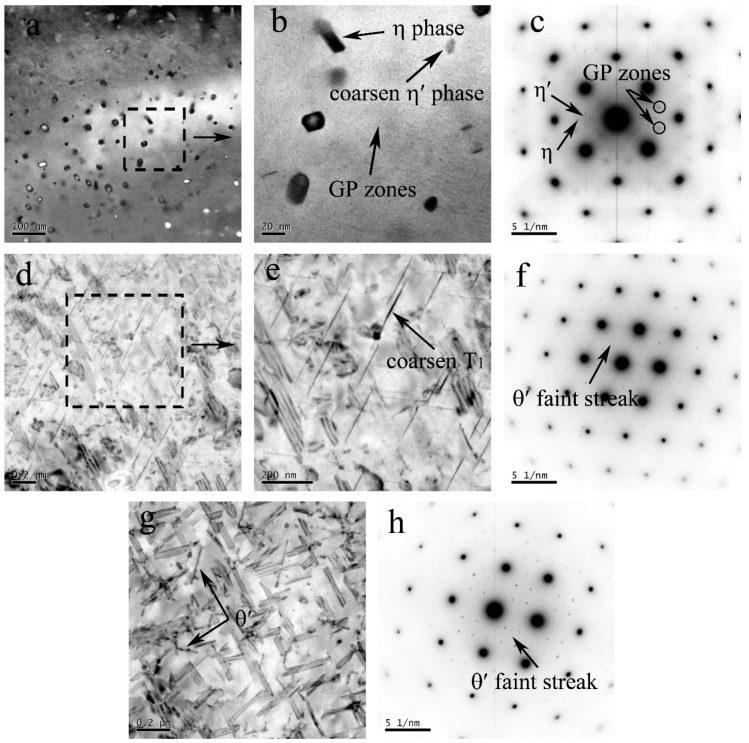
TEM observation of the inside of the grains for the different zones of the Al–Zn–Mg–Cu alloy at AS: BF-images of (**a**) HAZ; (**b**) high magnification of HAZ; and (**c**) SAD pattern of HAZ along [100]_Al_ axis zone; and for the different zones of the Al–Li–Cu alloy at RS: BF-images of (**d**) HAZ; (**e**) high magnification of HAZ; (**f**) SAD pattern of HAZ along [110]_Al_ axis zone; (**g**) BF-images of HAZ; and (**h**) SAD pattern of HAZ along [100]_Al_ axis zone.

Because of the heat effect, the temperature in HAZ could reach 400 °C, which led the structure of precipitates to transform. For the HAZ of the Al–Zn–Mg–Cu alloy, intragranular precipitates in the HAZ were characterized by the SAD pattern (Figure 6c) along the [100]_Al_ axis zone, which indicated those were mainly η′ phase and GP I zone. Compared with the microstructure of BM, η′ phase is severely coarsened and sparsely populated in the grains (Figure 6a,b) with a size of about 40 nm. It illustrated that the η′ phase was simultaneously coarsened and dissolved in HAZ during the thermal transient.

For the HAZ of the Al–Li–Cu alloy, compared with BM, the T_1_ precipitates in HAZ obviously is thicker, but the length has no obvious difference. The TEM-BF images (Figure 6d,e) show that the number of T_1_ precipitates decreased and that the θ′ precipitates almost disappeared, the dissolution of the θ′ phase is evidenced by the faint streaks corresponding to θ′ in the SAD pattern (Figure 6f) along the [110]_Al_ axis zone and the SAD pattern (Figure 6h) along [100]_Al_ axis zone. This phenomenon indicates that the T_1_ precipitates possess a higher thermal stability compared with the θ′ precipitates.

**Figure 7:**
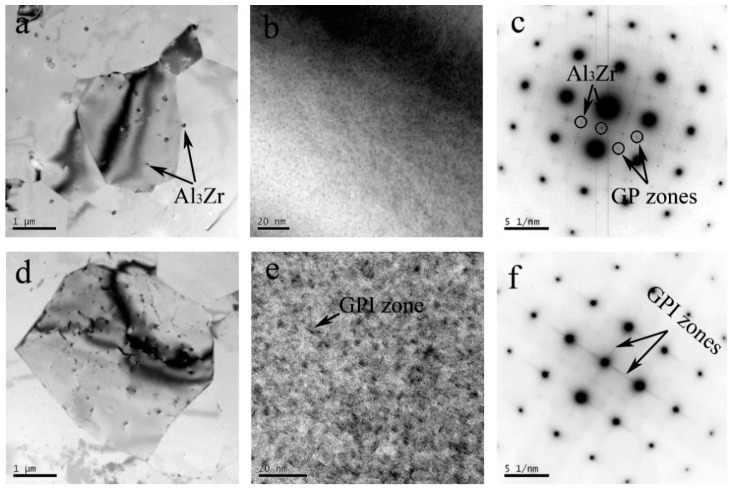
TEM observation for NZ of the Al–Zn–Mg–Cu alloy (**a**) and (**b**) corresponding to the SAD pattern (**c**) along the [100]_Al_ axis zone; TEM observation for the NZ of the Al–Li–Cu alloy (**d**); and (**e**) corresponding to the SAD pattern (**f**) along th e[100]_Al_ axis zone.

As a result of the severe plastic deformation and heat deriving from friction and stir, the highest temperature at NZ was over 450 °C, and the grain was equiaxed because of the dynamic recrystallization (Figure 7a,d). During the cooling and nature ageing process, GP zones formed in the Al–Zn–Mg–Cu alloy, as shown in Figure 7b. The SAD pattern (Figure 7c) also only evidences the existence of the GP zones and Al_3_Zr within the grains. The superlattice reflections of η′ and η were not observed, which indicates that η′ and η adequately dissolved during the weld process. Similarly, for the NZ of Al–Cu–Li alloy, the presence of δ′ phase and GPIzones are evidenced by the corresponding faint spots and streaks (Figure 7f). Many tiny GP I zones are directly detected in the BF images (Figure 7e), and their length is approximately only 4 nm. In addition, the superlattice reflections of the θ′ and T_1_ precipitates are absent, which represents those phases dissolved into the Al matrix. Compared with the SAD pattern of BM, we found that the δ′ precipitates only are present in NZ, and it might be limited by the sample position for the TEM test. The δ′ precipitates may exist somewhere inside the TMAZ or HAZ, because the T_1_ and θ′ precipitates are partially dissolved.

### 3.3. Microhardness of the Weld Zone

**Figure 8:**
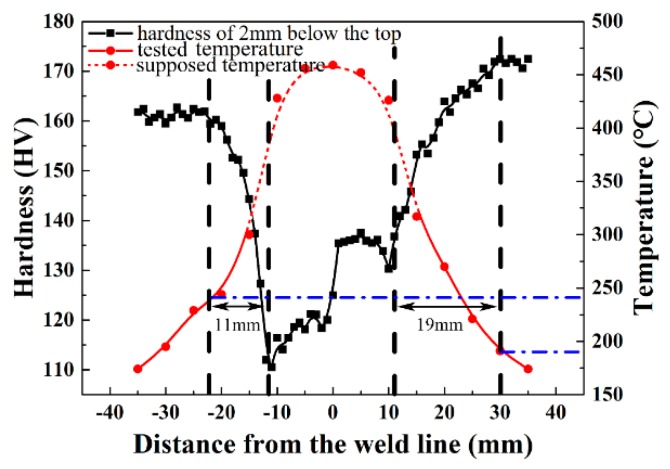
Microhardness distribution on a cross-section of the weld joint.

Figure 8 gives a microhardness profile on a cross-section of the weld joint 2 mm below the top. It does not exhibit a typical ‘W’ shape of a FSW joint. Base materials of the Al–Zn–Mg–Cu alloy possess the highest hardness value approximate was 172 HV, the highest hardness of the Al–Li–Cu alloy is 162 HV at BM. The minimum microhardness appears in the TMAZ of both AS and RS. The lowest microhardness of the Al–Zn–Mg–Cu alloy at AS was 123 HV, which was higher than that of the Al–Li–Cu alloy at RS, which was only 105 HV. It is interesting that the width of the HAZ at AS was obviously wider than that at RS.

The red curve in Figure 8 is the temperatures in different positions. The dash red line was the supposed temperatures because of a difficulty measuring, and the solid red line was the measured temperatures. The temperature gradually decreased from the center to the edges, and the decreasing rate of temperature also became slow.

### 3.4. Diffusion at Periphery of the Kissing Line

**Figure 9:**
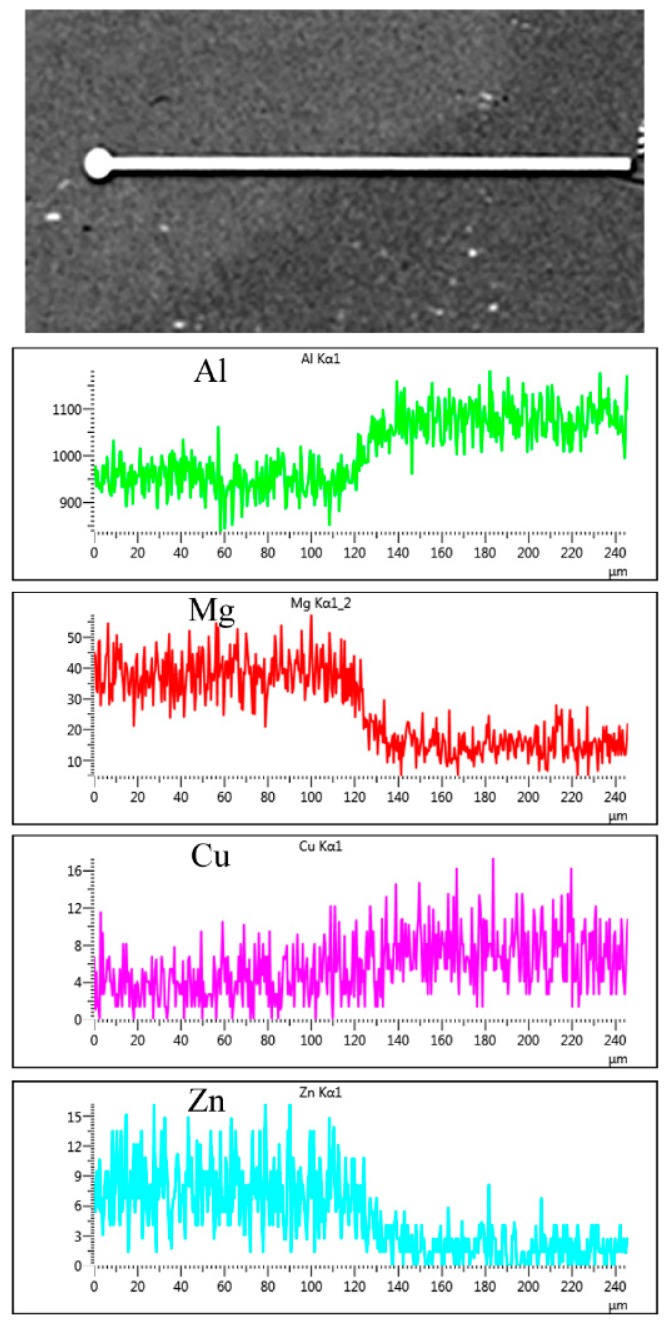
X-ray line scanning for the periphery of the kissing line.

Figure 9 displays the elements distribution at the periphery of the kissing line. The weld kissing line of two types of alloys obviously was observed by a backscattered electron. According to the result of the line scanning, it illustrates that only magnesium presented a remarkable diffusion, but the contents of copper and zinc did not show a gradual change process like magnesium. The diffusion coefficient of magnesium into aluminum is higher than copper and zinc into aluminum. The content of aluminum also showed a decrease in the Al–Li–Cu side because of the diffusion of magnesium from the Al–Zn–Mg–Cu alloy to the Al–Li–Cu alloy.

### 3.5. Corrosion Behavior

**Figure 10:**
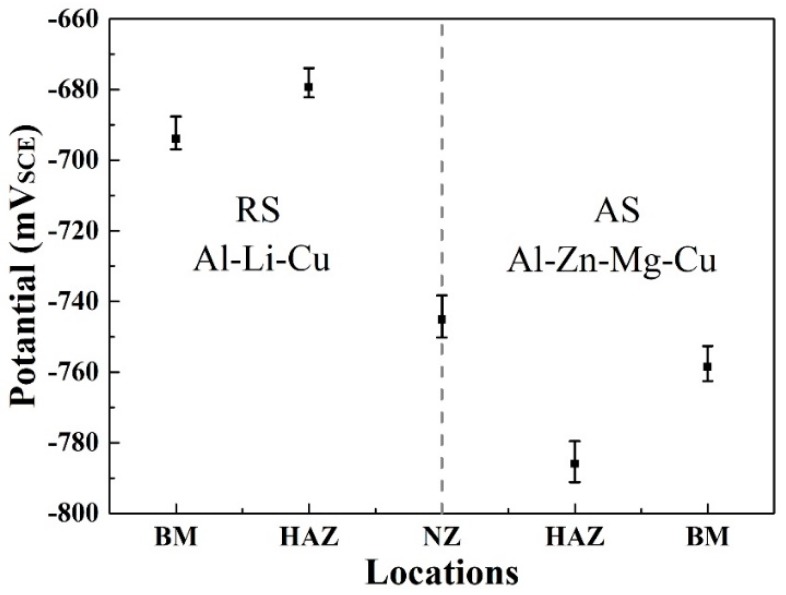
The open circuit potential (OCP) of the FSW joint Al–Zn–Mg–Cu alloy to the Al–Li–Cu alloy surface in a 3.5% NaCl solution.

**Figure 11:**
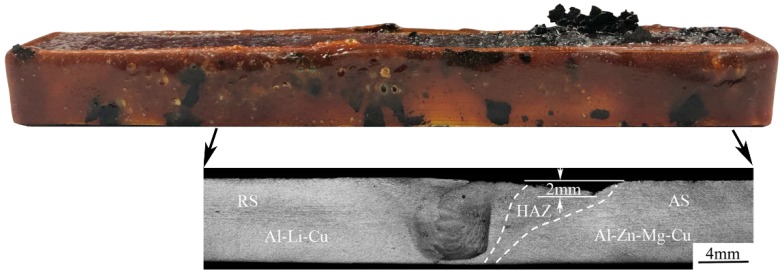
The corrosion morphology of joint through the exfoliation corrosion (EXCO) test.

The open circuit potential (OCP) of the various weld zones in the 3.5 wt % NaCl solution are displayed in Figure 10. Each of the zones was measured three times to ensure reproducibility. The HAZ at AS has the most negative OCP at −785 mV_SCE_. Additionally, the OCP of BM at AS is slight more positive than HAZ. For the Al–Li–Cu alloy, the HAZ and BM of OCP are −680 mV_SCE_ and −695 mV_SCE_, the HAZ is slight more positive than BM, both the HAZ and BM show a more positive OCP compared with the position at AS. Because of the difference in composition, the OCP of all of the zones of the Al–Zn–Mg–Cu alloy are always relatively negative compared with the Al–Li–Cu alloy. Therefore, it is expected that the HAZ of the Al–Zn–Mg–Cu alloy is the most susceptible to corrosion compared with the other welding zone. This is evidenced by the result of EXCO, as shown in Figure 11.

The interesting EXCO morphology for the weld joint is displayed in Figure 11. It is consistent with the microhardness profile, which was conducted 2 mm below the surface; the maximum EXCO depth, close to 2 mm, was observed in the HAZ of the Al–Zn–Mg–Cu alloy. The NZ of the Al–Zn–Mg–Cu alloy was exfoliated at a lower degree. But other zones at the AS present a better corrosion resistance. The EXCO result illustrated that there was a galvanic couple when the weld joint was exposed to a corrosive environment. The HAZ of the Al–Zn–Mg–Cu alloy were popular for attack, and other zones are protected because of the effect of the galvanic couple.

## 4. Discussion

The hardness of TMAZ is 10 HV lower than the NZ at AS, and there also is a difference of 20 HV between the TMAZ and NZ at RS. Because the grain size of the NZ was far finer than the TMAZ, as Figure 3 and Figure 4 show, fine-grain strengthening is one of the strengthening mechanisms for NZ, as the Hall-Petch formula [25] stated:

(1) $$\sigma_{s}=\sigma_{0}+kd^{-1/2}$$

where $\sigma_{s}$ is the yield strength, $\sigma_{0}$ and k are appropriate constants, and d is the diameter of grain. In addition, the NZ has a higher supersaturation of Zn, Mg, Cu, and Li atoms, because of all of the η′, θ′, and T_1_ precipitates dissolved into the Al matrix during weld process, which resulted in a greater nature ageing and solution strengthening. However, a higher degree of alloy in the NZ of the Al–Zn–Mg–Cu alloy (9.2%) resulted in a higher hardness.

In TMAZ, the highest temperature (>400 °C) was only slightly lower than the NZ during the weld process, most of the precipitates completely dissolved like the solution treatment. So, because of the loss of fine-grain strengthening and the lower strengthening from the GP zones, which are as a result of the nature ageing, the hardness of TMAZ was always lower than the NZ both in the AS and RS. The peak temperature in the HAZ was higher than 250 °C. A lower percentage of precipitates in the HAZ dissolved compared with the TMAZ. There are a number of GP zones forming during nature ageing. Another part of the precipitates was coarser, which was considered as an overaged condition, which maintained the effect of strengthening. The GP zones were present due to the subsequent nature ageing, as the Figure 5 shows. The strengthen effect of the η′, η, and T_1_ phase can be given by the formula [26],

(2) $$\Delta \sigma =0.12MG\frac{b}{2\sqrt{rh}}\left[f^{1/2}+0.70{\left(\frac{r}{h}\right)}^{1/2}f+0.12\left(\frac{r}{h}\right)f^{3/2}\right]ln\left(\frac{0.158r}{r_{0}}\right)$$

where *G* is the shear modulus of aluminum, *b* is the Burgers vector, f is the precipitates fraction, $f_{rex}$ is the recrystallized fraction, the *δ* is the recrystallized region (subgrain) grain size, *D* is the recrystallized grain size, and *α* is a constant. In Figure 6a–c, the coarsened η′ phase, η phase, and GP zones were observed. The strengthening effect coming from the GP zones cannot compensate the loss of strengthening due to the dissolved η′ phase, resulting in the hardness decreasing in the AS. Figure 6d,e indicate the θ′ phase dissolving and the T_1_ phase decreasing and coarsening at RS, it also means that the hardness decreases at HAZ. The precipitates fraction f decreasing lead to the hardness of the HAZ gradually increasing, which was away from the weld center, due to the heat accumulation decreasing.

The width of the HAZ for the Al–Zn–Mg–Cu alloy was 8 mm wider than that of the Al–Li–Cu alloy, but the thermal transmission range was absolutely symmetrical along the weld center during the weld process. The T_1_ precipitates are the dominant strengthening phase in Al–Li–Cu alloys when temperature was below 260 °C [27,28], but the dissolution temperature of the η′ phase in the Al–Zn–Mg–Cu alloys is approximately 180 °C [29]. The tested temperature was evidence of the phenomenon, as the red curve indicates in Figure 7. Therefore, the zone of the Al–Zn–Mg–Cu alloy did not suffer from the thermal transient as it was further away from the weld center.

**Figure 12:**
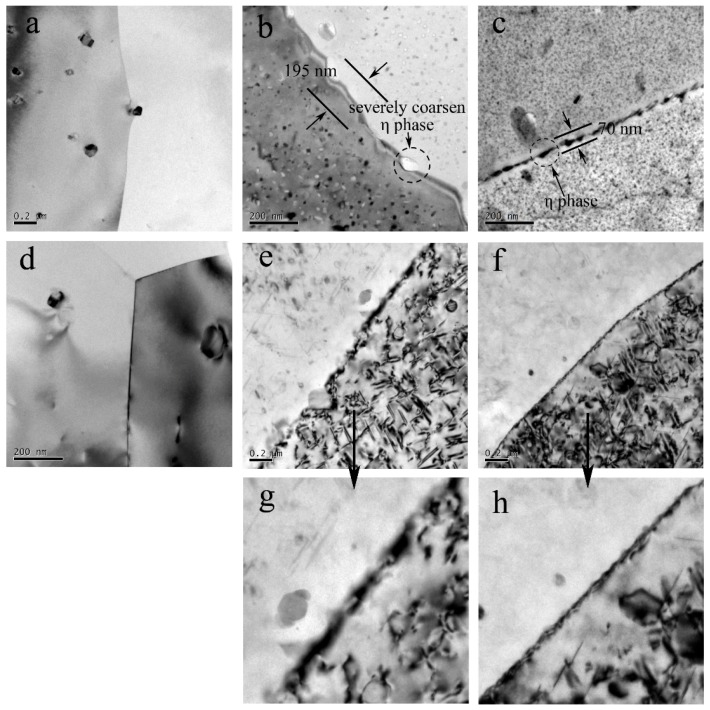
The grain boundaries of the Al–Zn–Mg–Cu alloy in: (**a**) the severely coarsened η phase and the widened PFZ in the HAZ; and (**b**) the smaller η phase and narrower PFZ in BM and (**c**) NZ, and the grain boundary of the Al–Li–Cu alloy in: (**d**) NZ; (**e**) HAZ; (**f**) BM; and (**g**) HAZ at an enhanced magnification (**h**) BM at an enhanced magnification.

The TEM micrographs for the grain boundaries are provided in Figure 12. For the Al–Zn–Mg–Cu alloy, the precipitates free zone (PFZ) width of the HAZ is almost 195 nm and the grain boundary is decorated by the severely coarsened η phase. Those coarsened η phases are discontinued, as indicated by the arrows in Figure 12b. However, the PFZ width of the BM with 70 nm is obviously narrower than that of HAZ, and the Figure 11c shows that the grain boundary precipitates are smaller in contrast to the HAZ, and the space of the adjacent particles is dramatically smaller. There is no PFZ in NZ because of the absence of precipitates, as Figure 12a shows. According to the previous research by Deng et al. [30], the addition of Zr and Sc in the Al–Zn–Mg alloy improved the stress corrosion cracking resistance and discouraged the exfoliation corrosion through restricting the precipitate free zone expanding and refining the grains. The PFZ as anode was compared to the Al matrix prior to corrosion. The wider the PFZ in the Al–Zn–Mg–Cu alloy, the more corrosion sensitivity will be shown in corrosive environments. This is why the HAZ of the Al–Zn–Mg–Cu alloy has a high corrosion susceptibility.

The grain boundaries of NZ are decorated by nothing, as shown in Figure 12a. So, it shows a more positive OCP compared with BM. However, there was no difference between the two zones on corrosion morphology, as Figure 11 shows. The HAZ are susceptible to localized corrosion and other welding zones are more resistant to corrosion. In the HAZ of the Al–Li–Cu alloy, the tiny T_1_ phase at the grain boundaries dissolved into Al matrix, as Figure 12e,g shown, but the interior T_1_ of the grains did not have a severe dissolution. In addition, Figure 12f,h present the T_1_ precipitates distributed on the grain boundaries in the BM, which results in grain boundaries at a more negative potential, because the T_1_ has a more negative potential (−1.096 VvsSCE) in contrast with the Al matrix (−0.7529 VvsSCE) [31]. Hence, the HAZ shows a more positive OCP compared with the BM at RS for the Al–Li–Cu alloy. However, they also present no difference in corrosion morphology due to the high susceptibilities of the HAZ of the Al–Zn–Mg–Cu.

## 5. Conclusions

(1) During the friction stir welding process, the original η′ phase precipitates in the HAZ of the Al–Zn–Mg–Cu alloy subjected to the thermal transient, coarsening, and dissolving process, occurred simultaneously. The GP zones appeared during the subsequent nature ageing. In the HAZ of the Al–Li–Cu alloy, the θ′ phase dissolved seriously, but most of the T_1_ precipitates only coarsened, and the density of the T_1_ precipitates slight decreased, because the thermal stability of the T_1_ precipitates is much more stable than the θ′ phase precipitates. Because of the nature ageing after welding, the NZ of the Al–Zn–Mg–Cu alloy only contains GP zones, and the NZ of the Al–Li–Cu alloy only contains GP Izones.
(2) During the friction stir welding process, the thermal that came from the NZ led to a different width of the HAZ for two types of alloys. The HAZ of the Al–Zn–Mg–Cu alloy is 7 mm wider than that of the Al–Li–Cu alloy. It is directly observed by the microhardness profile. Thus, the thermal stable temperature of the η′ phase precipitates in Al–Zn–Mg–Cu alloys is approximate to 180 °C, but that of T_1_ precipitates in Al–Li–Cu alloys is close to 260 °C.
(3) There is only a diffusion of the magnesium element at the periphery of the kissing line during the welding process. However, the content of copper and zinc have no change because of the low diffusion coefficient in the aluminum matrix.
(4) The HAZ of the Al–Zn–Mg–Cu alloy has the most negative open circuit potential compared with the other welding zones, and is responsible for the severely localized corrosion. Additionally, all of the zones of the Al–Li–Cu alloy show a good corrosion resistance and have a more positive potential compared with the Al–Zn–Mg–Cu ally.
